# A Uranium-Based UO_2_^+^–Mn^2+^ Single-Chain Magnet Assembled trough Cation–Cation Interactions**

**DOI:** 10.1002/anie.201307366

**Published:** 2013-12-06

**Authors:** Victor Mougel, Lucile Chatelain, Johannes Hermle, Roberto Caciuffo, Eric Colineau, Floriana Tuna, Nicola Magnani, Arnaud de Geyer, Jacques Pécaut, Marinella Mazzanti

**Affiliations:** Laboratoire de Reconnaissance Ionique et Chimie de Coordination SCIBUMR-E3 CEA-UJF, INAC, CEA-Grenoble, 17 rue des Martyrs, 38054 Grenoble Cedex 09 (France); Service Général des Rayons XSP2M, INAC, CEA-Grenoble, 17 rue des Martyrs, 38054 Grenoble Cedex 09 (France); EPSRC UK EPR FacilitySchool of Chemistry and Photon Science Institute, The University of Manchester, Oxford Road, Manchester, M13 9PL (UK); European CommissionJoint Research Centre, Institute for Transuranium Elements, P.O. Box 2340, 76125 Karlsruhe (Germany); Institute of NanotechnologyKarlsruhe Institute of Technology, Hermann-von-Helmholtz Platz 1, 76344 Eggenstein-Leopoldshafen (Germany)

**Keywords:** actinides, cations, polymetallic complexes, single-chain magnets, uranium

## Abstract

Single-chain magnets (SCMs) are materials composed of magnetically isolated one-dimensional (1D) units exhibiting slow relaxation of magnetization. The occurrence of SCM behavior requires the fulfillment of stringent conditions for exchange and anisotropy interactions. Herein, we report the synthesis, the structure, and the magnetic characterization of the first actinide-containing SCM. The 5f–3d heterometallic 1D chains [{[UO_2_(salen)(py)][M(py)_4_](NO_3_)}]_*n*,_ (M=Cd (**1**) and M=Mn (**2**); py=pyridine) are assembled trough cation–cation interaction from the reaction of the uranyl(V) complex [UO_2_(salen)py][Cp*_2_Co] (Cp*=pentamethylcyclopentadienyl) with Cd(NO_3_)_2_ or Mn(NO_3_)_2_ in pyridine. The infinite UMn chain displays a high relaxation barrier of 134±0.8 K (93±0.5 cm^−1^), probably as a result of strong intra-chain magnetic interactions combined with the high Ising anisotropy of the uranyl(V) dioxo group. It also exhibits an open magnetic hysteresis loop at *T*<6 K, with an impressive coercive field of 3.4 T at 2 K.

Single-chain magnets (SCMs) present an attractive alternative to discrete molecular clusters behaving as single molecule magnets (SMMs) in the design of molecular materials for magnetic information storage and processing.^1^ SCMs^2^ are one-dimensional coordination polymers that display slow relaxation of the magnetization and hysteresis effects as a result of the intra-chain exchange interactions that usually develop into 1D ferromagnetic spin–spin correlations at low temperature. In the design of improved SCMs required for application at practical temperatures, three strict requirements need to be fulfilled: a strong Ising anisotropy of the magnetic centers, strong intra-chain magnetic interactions, and weak interchain interactions. Since the first experimental evidence of the existence of a SCM was reported in 2001^3^ (predicted earlier by Glauber^4^), efforts in the design of SCMs with higher reversal barriers have focused on the use of metal ions with strong anisotropy, such as Co^2+^, Ni^2+^, Mn^3+^, Fe^2+^, Re^4+^,2c, 5 and, more recently, lanthanide ions.^6^

Actinides, and uranium in particular, are currently attracting large attention in the field of molecular magnetism because of their large single-ion anisotropy and enhanced covalency, as compared to lanthanide ions, which should promote magnetic communication.^7^ As such, uranium-based compounds are well suited for the design of molecular magnets with higher anisotropy barriers and hysteresis temperatures for practical applications. Several examples of mononuclear complexes of uranium showing slow relaxation of magnetization have been reported in the last few years.^8^ The single-ion magnetic behavior of these compounds arises from the high anisotropy generated by the axial ligand environment. Fewer examples of polynuclear-actinide-based single-molecule magnets have also been reported.^9^ However, to date there are no reported examples of actinide-based SCMs.

Cation–cation interactions^10^ (CCI; a term used to describe the bonding interaction of an actinyl oxo or imido group with a metal cation) provide a versatile route for the assembly of homopolymetallic and heteropolymetallic discrete clusters9c, 11–13, 17 or 1D chains^14^ of pentavalent uranium, and a pathway for intermetallic magnetic exchange.9c, 12, 13a, 15 We have also recently reported the first 5f–3d cation–cation cluster, a large U_12_Mn_6_ wheel that exhibits SMM behavior,9c but CCI has not yet been used to promote the assembly of 1D chains associating pentavalent uranyl and d-block transition metals.

Herein, we report the first example of a uranium-based SCM that is formed by CCI between the Mn^II^ ion and the two oxo groups of a uranyl(V) complex. This infinite chain displays a high relaxation barrier of 134±0.8 K, probably as a result of strong intra-chain magnetic interactions combined with the high Ising anisotropy of the uranyl(V) dioxo group. It also exhibits an open magnetic hysteresis loop at *T*<6 K, with an impressive coercive field of 3.4 T at 2 K.

The reaction of the monomeric uranyl(V) complex [UO_2_(salen)py][Cp*_2_Co] with Cd(NO_3_)_2_ in pyridine in a 1:1 ratio affords the coordination polymer [{[UO_2_(salen)(py)][Cd(py)_4_](NO_3_)}]_*n*_ (**1**), as a pink microcrystalline powder in 65 % yield (Scheme 1). X-ray quality single crystals of **1**⋅2 py were obtained by slow diffusion of pyridine solutions of the two reactants. Using a similar procedure, the manganese analogue [{[UO_2_(salen)(py)][Mn(py)_4_](NO_3_)}]_*n*_ (**2**) was synthesized in 65 % yield.

**Scheme 1:**
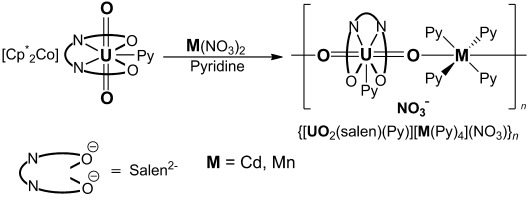
Synthesis of the 1D chains 1 and 2.

Both complexes are stable in the solid state for months under argon atmosphere. It is also quite remarkable that, in spite of the higher charge of the Mn^2+^ and Cd^2+^ ions compared to UO_2_^+^, scrambling of the salen ligand is not observed, which points to the presence of a very strong CCI interaction in **1** and **2**.

X-ray diffraction studies of **1** show the presence of alternating layers of NO_3_^−^ anions and of cationic dimetallic chains {[UO_2_(salen)(py)][Cd(py)_4_]}_*n*_^*n*+^ (Figure 1; see also the Supporting Information, Figure S2). The asymmetric unit of **1** contains three uranium and three cadmium ions, which are crystallographically non-equivalent due to the non-linear arrangement of the UO_2_^+^ groups and Cd^2+^ ions along the chain (Figure 1, bottom). The cationic polymeric chain {[UO_2_(salen)(py)][Cd(py)_4_]}_*n*_^+^ is formed by the cation–cation interaction of each uranyl(V) oxo group of [UO_2_(salen)py]^−^ complexes with a Cd^2+^ ion. The U-O-Cd angle deviates slightly from linearity and ranges from 161.67° to 175.15°. The uranium atom is heptacoordinated with a slightly distorted pentagonal bipyramidal geometry, with the four donor atoms of the salen ligand situated in the equatorial plane and the two uranyl oxygens in the axial position; the seventh coordination position is occupied by a pyridine nitrogen. The cadmium ion is six coordinated in an octahedral geometry, with the two uranyl(V) oxo groups in apical positions and the four pyridine nitrogens in its equatorial plane. The mean Cd–O_yl_ distance of 2.28(2) Å, is in the range of those found in a heterobimetallic U^VI^/Cd^II^ carboxyphosphonate networks with Cd^2+^ ions coordinated to the apical oxygens of the uranyl(VI) moieties^16^ (Cd-O_yl_=2.252(4) Å). The U–O_yl_ distance in **1** (1.87(2) Å) is in the range of U–O_yl_ distances found for uranyl(V) oxo groups involved in cation–cation interactions leading to discrete clusters9c, 17 or 1D polymeric chains.^14^, ^18^

**Figure 1:**
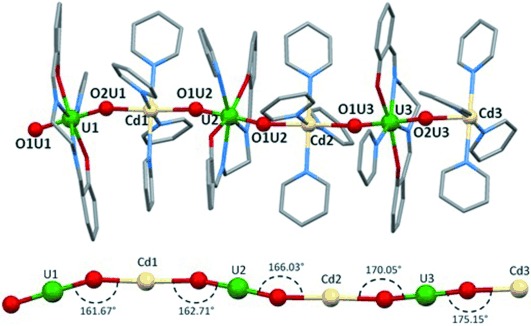
Mercury view of the structure of 1 (top) and a detail of the core with associated distances and angles (bottom). Hydrogen atoms and cocrystallized solvent molecules omitted for clarity. C grey, O red, Cd cream, N light blue, U green.

X-ray analysis was also performed on single crystals of **2** and shows the presence of a coordination polymer isostructural to complex **1** (see the Supporting Information). The poor quality of the crystals does not lead to a publishable structure, but the connectivity of the polymer is unambiguously determined. The difference in ionic radii of Mn^2+^ (0.67 Å) compared to Cd^2+^ (0.95 Å) results in shorter intra-chain separations between neighboring U^V^ ions (U–U=8.0 and 8.1 Å in **2**, and 8.19 and 8.36 Å in **1**) and between neighboring M^II^ ions (Mn–Mn=8.1 Å in **2**, and Cd–Cd=8.32 and 8.25 Å in **1**).

X-ray powder diffraction patterns recorded for microcrystalline samples of **1** and **2** (see the Supporting Information) are consistent with those calculated from the X-ray single crystal data and show that both bulk samples contain homogeneous isostructural compounds.

There is no evidence of significant inter-chain hydrogen bonding or π-stacking interactions in the structure of **1**. Owing to the presence of the bulky salen ligand, the chains are well-separated, with the shortest inter-chain U–U and U–Cd distances at 11.99 and 11.69 Å, respectively, in **1**; the shortest inter-chain U–U, U–Mn and Mn–Mn distances are 11.4, 10.9 and 11.5 Å, respectively, in **2**. These features indicate the presence of magnetically isolated chains in the two isostructural complexes **1** and **2**.2a,c

Variable-temperature (2–300 K) magnetic susceptibility measurements were performed on polycrystalline samples of **1** and **2** in static magnetic fields ranging from 0.01 to 5 T (Figure 2 and the supplementary information). The measured *χ* *T* value for **2** at room temperature is approximately 4.3 cm^3^ K mol^−1^; considering that the susceptibility curves for the Cd-based analogue **1** (see the Supporting Information) point towards a much smaller *χ* *T* value (below 0.3 cm^3^ K mol^−1^) we can conclude that this value is in line with what is expected for one spin-only divalent manganese (with *S*=5/2 and g close to 2) and one pentavalent uranium ion, whose magnetic moment is significantly reduced with respect to the free-ion value by the combined effect of ligand field and covalent bonding.^19^ The *χ* *T* product decreases with decreasing temperature to 4.1–4.2 cm^3^ K mol^−1^ at 150 K; the fact that the same quantitative behavior is observed for **1** and that the decrease is similar in absolute value for the two compounds, allows the attribution of this effect to the ligand-field state depopulation for the anisotropic uranium centers, whereas the contribution of the more isotropic manganese ions can be approximately regarded as constant within this temperature range. Below 150 K, the susceptibility of **2** increases to reach a field-dependent maximum, with values of 56.8 cm^3^ K mol^−1^ at 0.01 T (Figure S9) and 52.7 cm^3^ K mol^−1^ at 0.05 T (Figure 2), before dropping rapidly at very low temperatures owing to saturation effects, magnetic anisotropy, and possibly inter-chain antiferromagnetic interactions. The increase of *χ* *T* below 150 K, as well as the strong deviation from the Curie–Weiss behavior of *χ*^−1^ vs. *T* (see the Supporting Information), suggests dominant ferromagnetic interactions leading to an aligned-spin ground state. None of this is observed for the Cd-based analogue **1**, where only an abrupt decrease of the *χ* *T* product below 25 K is observed, which is most likely due to single-ion crystal field effects associated with U^V^,8a quenching of the orbital angular momentum, and possibly weak next-nearest-neighbor antiferromagnetic exchange between uranium centers.

**Figure 2:**
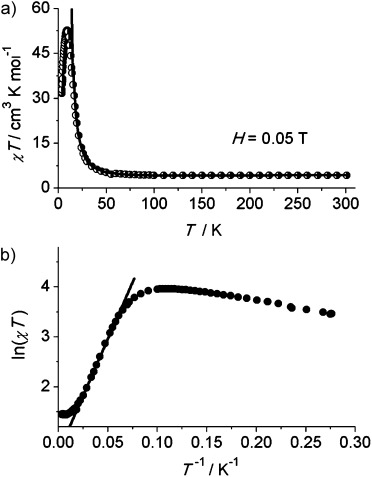
Plots of a) χ*T* vs. *T* and b) ln (*χ* *T*) vs. 1/*T* for a polycrystalline sample of 2 measured at 0.05 T applied field.

A scaling procedure of the *χ* *T* data of **2** (Figure 2) clearly indicates the occurrence of a linear regime, which is characteristic of Ising 1D systems.2a, 20 The ln(*χ* *T*) versus 1/*T* plot increases linearly between 45 and 16 K. Fitting the experimental data within this linear regime using the equation *χ* *T*=*C*_eff_ exp(Δ/*k*_B_ *T*), which describes a ferromagnetically coupled infinite chain, gives an energy gap (Δ/*k*_B_) of 45.5 K and a pre-exponential factor (*C*_eff_) of 1.98. Very similar results for the ferromagnetic exchange gap are obtained by fitting the magnetic susceptibility data of **2** at 16–300 K with the equation *χ* *T*=[*C*_1_ exp(Δ_1_/*k*_B_ *T*)]+[*C*_2_ exp(Δ_2_/*k*_B_ *T*)], where a second negative exponential that vanishes at 0 K is added to take into account the high-temperature crystal field effect or antiferromagnetic contribution.6c In this case, we obtained Δ_1_/*k*_B_=45.5 K, Δ_2_/*k*_B_=−90.2±9.4 K, *C*_1_=1.98, and C_2_=2.73, which is in very good agreement with the previous considerations. As expected, the high-temperature extrapolated Curie constant, *C*−*C*_1_+*C*_2_=4.71 cm^3^ K mol^−1^, is close to the expected value for one Mn^II^ ion and one U^V^ ion.

The existence of a magnetic ground state in **2** is further confirmed by the observation of magnetic hysteresis loops. As shown in Figure 3, magnetic bistability is observed in all magnetization versus field scans at 2–5 K. With decreasing temperature, the coercive field increases, reaching a value of 3.4 T at 2 K. At zero field, a remanent magnetization (REM) of 1.7 μ_B_ is preserved. This behavior is typical of a single-chain magnet below its blocking temperature (*T*_B_). Indeed, below 6 K a divergence is observed between zero-field-cooled and field-cooled magnetizations as a function of temperature (see the Supporting Information). In addition, REM vanishes at ca. 5.8 K, which corresponds to the blocking temperature of the material.

**Figure 3:**
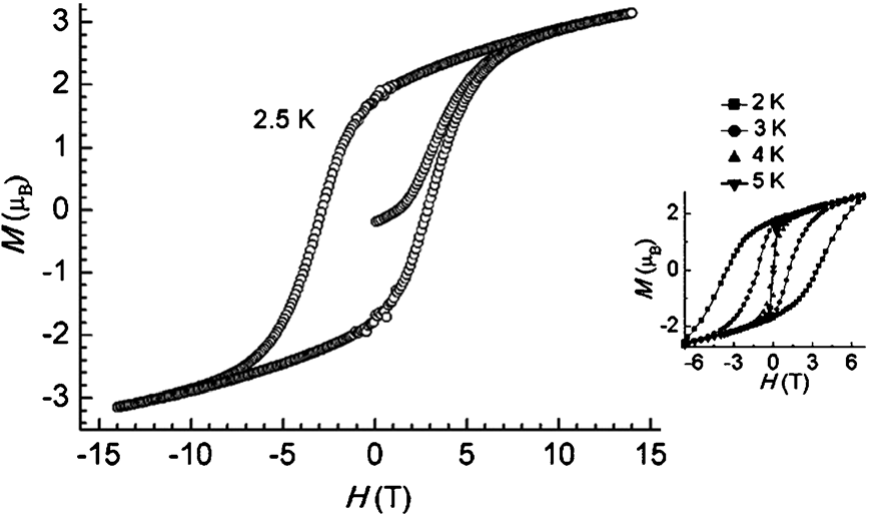
Field dependence of the magnetization of 2 measured at 2.5 K. Inset shows hysteresis loops recorded at four different temperatures.

To probe the magnetization relaxation in **2**, zero-field alternating current (AC) susceptibility measurements at 2–15 K were carried out at several frequencies: at 10–9887 Hz with a 10 G AC field (Figure 4; see also the Supporting Information), and at 0.1–1399 Hz with a 1.55 G AC field (see the Supporting Information). Below 12 K, both the in-phase (*χ*′) and out-of-phase (*χ*′′) components of the AC susceptibility are strongly frequency dependent, and *χ*′(*T*,*f*) and χ′′(*T*,*f*) maxima are clearly observed (*f* is the AC frequency). This result precludes any tri-dimensional ordering; moreover, the relative variation of the temperature of the *χ*′′ peak with respect to the frequency is measured by a parameter *ϕ*=(Δ*T*_max_/*T*_max_)/Δ(log *f*)=ca. 0.13, which is in the range of normal superparamagnets, and excludes the possible occurrence of a spin glass state.15a, 21

**Figure 4:**
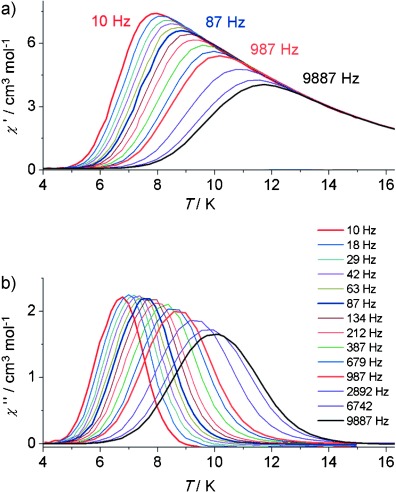
Temperature dependence of the a) real (*χ*′) and b) imaginary (*χ*′′) AC susceptibility for 2 measured at zero static field and 10 G AC field.

Semicircular Cole–Cole plots (*χ*′′ vs. *χ*′) are obtained for temperatures below 10 K, which can be fitted to a generalized Debye model^22^ with an *α* parameter of 0.20–0.43; this is indicative of a moderately wide distribution of relaxation times (see the Supporting Information). The magnetization relaxation time obtained from the AC experiments as a function of temperature and frequency was fitted to the Arrhenius equation *τ*=*τ*_0_ exp(Δ*E*/*k*_B_*T*), where τ is the relaxation time, Δ*E* is the energy barrier for the relaxation of magnetization, and τ_0_ is the pre-exponential factor (Figure 5). From the least-squares fit, Δ*E* was found to be 134±0.8 K (93±0.5 cm^−1^) and *τ*_0_=3.1×10^−11^ s. As expected, the Δ*E* barrier extracted from the AC data is larger than the energy gap deducted from susceptibility measurements, a situation that is often observed in SCMs, particularly those consisting of highly-anisotropic repeating units.2a, 5a In such cases, the dynamics of the magnetization are governed by both magnetic correlations and the relaxation barrier experienced by individual magnetic units.^20^ The large anisotropy of **2** is explained by the strong Ising-type ligand field due to the close pair of linearly arranged oxygens characteristic of the uranyl group.19b A similar situation occurs in **1** and indeed slow relaxation of the magnetization due to anisotropic U^V^ units is observed at low temperatures, under applied field (see the Supporting Information). SMM behavior in a monometallic U^V^ terminal mono-oxo complex was recently reported by Liddle et al.8a The polymeric chain **2** is the first example of an actinide-based SCM. Its thermal relaxation barrier of 134 K (93 cm^−1^) is slightly smaller than that of the previously reported U_12_Mn_6_ SMM (Δ*E*= ca. 142 K (99 cm^−1^)),9c but significantly larger than those reported for lanthanide-based single-chain magnets.^2^, ^6^ Lower values of the relaxation energy barrier were reported for SMMs based on mononuclear U^III^ and U^V^ (highest value: 30 K (21 cm^−1^)).^8^ Moreover, compound **2** shows the largest blocking temperature ever reported for any actinide-based molecular magnet.

**Figure 5:**
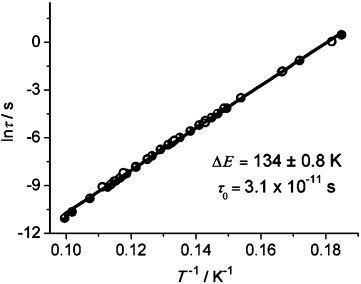
Arrhenius plot displaying T-dependence of the relaxation times for 2. Open circles indicate that the corresponding relaxation time was extracted from fitting the frequency-dependent AC susceptibility curves with a modified Debye model (see the Supporting Information), whereas the dots indicate that the temperature corresponding to the peak maximum in AC curves was measured at constant frequency.

In conclusion, we have shown that 5f–3d heterometallic 1D chains can be conveniently built taking advantage of the strong cation–cation interaction occurring between the pentavalent uranyl oxo groups and Cd^II^ or Mn^II^, which prevents scrambling of the salen ligand. The Mn-UO_2_-Mn coordination polymer exhibits a slow relaxation of magnetization with a high relaxation barrier and shows an open hysteresis, thus providing the first example of an actinide-based SCM. The high magnetic anisotropy of the pentavalent uranyl complex and the high spin of Mn^II^ associated with significant intra-chain magnetic communication and long interchain intermetallic distances are probably at the origin of the SCM behavior. The convenient route to uranium-based 1D heterodimetallic chains presented here, in association with the wide range of possible Schiff bases available, provides an entry to the development of actinide-based SCMs.
