# Supplementary material for: A Uranium-Based UO2+–Mn2+ Single-Chain Magnet Assembled trough Cation–Cation Interactions
Source: Angew Chem Int Ed Engl. 2013 Dec 6;53(3):819–23. doi: 10.1002/anie.201307366 (PMC4232274; doi:10.1002/anie.201307366)
Supplement: Supplementary file 1 [file anie0053-0819-sd1.pdf]

Angewandte Chemie
Eine Zeitschrift der Gesellschaft Deutscher Chemiker

Supporting Information

© Wiley-VCH 2013

69451 Weinheim, Germany

A Uranium-Based UO_2^+–Mn^{2+} Single-Chain Magnet Assembled trough Cation–Cation Interactions**

*Victor Mougel, Lucile Chatelain, Johannes Hermle, Roberto Caciuffo, Eric Colineau, Floriana Tuna, Nicola Magnani, Arnaud de Geyer, Jacques Pécaut, and Marinella Mazzanti**

anie_201307366_sm_miscellaneous_information.pdf

Table of Contents :

1. **General considerations.** ... 3
2. **Synthesis.** ... 4
 2.1 Synthesis of {[UO_2(salen)py][Cd(py)$_4$]NO_3}$_n$, 1. ... 4
 2.2 Synthesis of {[UO_2(salen)py][Mn(py)$_4$]NO_3}$_n$, 2. ... 4
3. **X-ray Crystallography.** ... 5
 Figure *S1*. Mercury view of the coordination environment of U1 and Cd1 in **1** with thermal ellipsoids at the 50% probability level. Hydrogen atoms were omitted for clarity ... 6
 Table *S1*. Crystallographic data of **1** ... 6
 Figure *S2* Cell packing of complex **1**.2Py showing the alternating layers formed by the cationic polymeric chains and the nitrate anions (cocrystallized pyridine are removed for clarity). ... 7
 Figure *S4*. Cell packing along the a axis of complex **1**.2py (cocrystallized pyridine and nitrate anions are removed for clarity). ... 9
 Figure *S5*. Mercury ball and sticks view of **2.** X-ray analysis shows that complex 2 is isostructural with complex **1** (cell parameters: 20.402(2); 21.776(3); 33.642(5); 90; 99.85(1); 90 ; space group $P2_1/c$). The high R value of 13.8% (due to the poor crystal quality) does not allow a detailed discussion of metrical parameters. ... 9
4. **Small angle powder X-Ray diffractograms.** ... 10
 Figure *S6*. Small angle X-ray diffractogram of bulk compound **1** {[UO_2(salen)py][Cd(py)$_4$]NO_3}$_n$3.2(py) (red line) and **2** {[UO_2(salen)py][Mn(py)$_4$]NO_3}$_n$0.5(py) (green line). ... 10
 Figure *S7*. Small angle X-ray diffractogram of bulk compound **1** {[UO_2(salen)py][Cd(py)$_4$]NO_3}$_n$3.2(py) (red line) and diffractograms calculated from single crystal structures **1** {[UO_2(salen)py][Cd(py)$_4$]NO_3}$_n$.2(py) (blue line). ... 11
 Figure *S8*. Small angle X-ray diffractogram of bulk compound **2** {[UO_2(salen)py][Mn(py)$_4$]NO_3}$_n$.0.5(py) (green line) and diffractograms calculated from single crystal structures of **2** (black line). ... 11
5. **Magnetic studies.** ... 11
 Figure *S9*. (left panel) Temperature dependence of χT for **2** measured at five different fields between 0.01 and 5 T. Inset: χ against T from 2 to 90 K measured at similar fields. (right panel) Temperature dependence of the magnetic susceptibility χ as a function of temperature T for **1** measured at three different fields between 0.5 and 5 T. Inset: Temperature dependence of χT for the same fields. ... 12
 Figure *S10*. Temperature dependence of $1/\chi$ for **2** measured at 0.01 T. ... 12
 Figure *S11*. Temperature dependence of the ac magnetic susceptibility for **2** measured at zero dc field and an ac field of 1.55 G oscillating at frequencies between 0.1 and 1400 Hz. ... 13
 Figure *S12*. Frequency dependence of the ac magnetic susceptibility for **2** measured at zero dc field and an ac field of 1.55 G oscillating at frequencies between 0.1 and 1400 Hz. ... 14
 Figure *S13*. Cole-Cole plots for **2** measured at zero-dc field and an ac field of 10 G oscillating at frequencies between 10 and 10000 Hz. ... 15
 Figure *S14*. Cole-Cole plots for **2** measured at zero-dc field and an ac field of 10 G oscillating at frequencies between 10 and 9987 Hz. ... 15
 Figure *S15*. Data (dots) and fits (lines) for the ac susceptibility curves of **2** measured at 7 (blue), 8 (green) and 9 K (red). The modified Debye model described in detail in Ref. 9(c) has been used. The extracted values of the relaxation times τ for the three given temperatures are 7.0×10^{-3} s, 6.8×10^{-4} s, and 1.1×10^{-4} s respectively; the exponents α, which describe the relaxation time distribution, is 0.43, 0.39, and 0.33 respectively. The curves have been measured at zero-dc field and an ac field of 10 G oscillating at frequencies between 10 and 10000 Hz. ... 15
 Figure *S16*. Temperature dependence of the zero-field cooled (ZFC), field-cooled (FC) and remanant (REM) magnetizations of **2** measured at 0.01 T. ... 16
 Figure *S17*. Frequency dependence of the (**left**) in-phase and (**right**) out-of-phase ac suspectibilities of **1** measured at 0.1 T dc field and 1.55 G ac field 21
 Figure *S18*. (Left) Cole Cole plots for **1** at temperatures between 1.8 amd 2.2 K; (right) Arrhenius plot displaying T-dependence of the relaxation time for **1**. ... 16

1. General considerations.

All manipulations were carried out under an inert argon atmosphere using Schlenk techniques and an MBraun glovebox equipped with a purifier unit. The water and oxygen level were always kept at less than 1 ppm. The solvents were purchased from Aldrich in their anhydrous form conditioned under argon and were vacuum distilled from K/benzophenone (hexane, pyridine) or CaH_2 (CH_2Cl_2). Depleted uranium turnings were purchased from the "Société Industrielle du Combustible Nucléaire" of Annecy (France). $Mn(NO_3)_2(Py)_3$ and $Cd(NO_3)_2(Py)_3$ were obtained by extraction of the hydrated salts in hot pyridine followed by high vacuum drying at 40°C for 7 days. Cp^*_2Co was purchased from Aldrich and sublimed prior to use. [UO_2(salen)(Py)] was synthesized as previously described.[1] Elemental analyses were performed under argon by Analytische Laboratorien GMBH at Lindlar, Germany. FTIR spectra were recorded with a Perkin Elmer Spectrum 100 Series FTIR spectrophotometer.

Elemental analyses were performed under argon by Analytische Laboratorien GMBH at Lindlar, Germany.

Caution: Depleted uranium (primary isotope ^{238}U) is a weak α-emitter (4.197 MeV) with a half-life of 4.47×10^9 years. Manipulations and reactions should be carried out in monitored fume hoods or in an inert atmosphere glovebox in a radiation laboratory equipped with α- and β-counting equipment.

[1] aV. Mougel, P. Horeglad, G. Nocton, J. Pecaut, M. Mazzanti, *Chem. Eur. J.*, *16*, 14365-14377; bV. Mougel, P. Horeglad, G. Nocton, J. Pecaut, M. Mazzanti, *Angew. Chem. Int. Ed.* **2009**, *48*, 8477-8480.

2. Synthesis.

2.1 Synthesis of {[UO_2(salen)py][Cd(py)$_4$]NO_3}$_n$, 1.

To a stirred orange suspension of [UO_2(salen)py] (100 mg, 0.16 mmol, 1 eq.) in 1 mL of pyridine is added a dark brown suspension of Cp^*_2Co (53.5 mg, 0.16 mmol, 1 eq.) in 1 mL of pyridine. The resulting dark green solution of [UO_2(salen)py][Cp^*_2Co] is then stirred for half an hour. A colorless solution of $Cd(NO_3)_2$ (38.4 mg, 0.16 mmol, 1 eq.) in 3 mL pyridine is then added, resulting immediately in a color change from green to dark violet. The solution is stirred for ten minutes at room temperature and then filtered. After letting the solution stand at room temperature overnight, a violet microcrystalline solid formed. This solid is filtered and washed with 10 x 1 mL pyridine until all traces of $Cp^*_2CoNO_3$ are removed. The solid is let drying few minutes in argon atmosphere (141 mg, 65 %).

Elemental analysis calcd (%) for **1**.3.2py, {[UO_2(salen)py][Cd(py)$_4$]NO_3}$_n$3.2(py) ($C_{57}H_{55}N_{11.2}O_7CdU$, M_r=1359.05) C 50.38, H 4.08, N 11.54; found C 50.34, H 4.17, N 11.55.

X-ray quality crystals of {[UO_2(salen) py][Cd(py)$_4$](NO_3)}$_n$ were obtained by slow diffusion in an H tube, where the solution of [UO_2(salen)py][Cp_2^*Co] (0.10 mmol, 1 eq.) in pyridine (4 mL) and the solution of $Cd(NO_3)_2(py)_3$ (0.10 mmol, 1 eq.) in pyridine (4 mL) were introduced in the two sections of the H tube connected by a layer of pyridine (10 mL). After two weeks diffusion; pink cubic crystals of {[UO_2(salen)(py)][Cd(py)$_4$](NO_3)}$_{n.}$ 2 py, **1.**2py, suitable for X ray analysis formed at the interface. A crystal of {[UO_2(salen)py][Cd(py)$_4$](NO_3)}$_{n.}$2py **1.** 2 py, collected directly from the solution was analyzed by X-ray diffraction. The remaining pink crystals were collected by filtration, washed with pyridine (3 x 1.5 mL) and quickly dried under vacuum to yield 68 mg of pink microcristallin solid (0.056 mmol, 56 %).

Elemental analysis(%) calculated for [{[UO_2(salen) py][Cd(py)$_4$](NO_3)}]$_n$·1.5(py) ($C_{48.5}H_{46.5}N_{9.5}CdO_7U$ 1224.65 g/mol) C 47.57, H 3.83 and N 10.87, found C 47.55, H 4.04 and N 10.96.

2.2 Synthesis of {[UO_2(salen)py][Mn(py)$_4$]NO_3}$_n$, 2.

To a stirred orange solution of [UO_2(salen)py] (100 mg, 0.16 mmol, 1 eq.) in 1mL of pyridine is added a dark brown suspension of Cp^*_2Co (53.5 mg, 0.16 mmol, 1 eq.) in 1 mL of pyridine. The dark green solution of [UO_2(salen)py][Cp^*_2Co] is then stirred for half an hour. A white suspension of $Mn(NO_3)_2(py)_4$ (67.6 mg, 0.16 mmol, 1 eq.) in 4 mL of pyridine is added to the resulting solution affording a red solution. After ten minutes the resulting solution is filtered and then left standing at room temperature overnight. A purple microcrystalline powder forms which is collected by filtration. This powder which is highly

insoluble in organic solvents was washed with 10 x 1 mL pyridine until all traces of $Cp*_2CoNO_3$ are removed and then dried under vacuum yielding complex **2**.0.5 py (114.1 mg, 66% yield).

Elemental analysis calcd (%) for **2**.0.5 py, $\{[UO_2(salen)py][Mn(py)_4]NO_3\}_n$.0.5py ($C_{43.5}H_{41.5}N_{8.5}O_7MnU$, M_r=1088.28) C 48.01, H 3.84, N 10.94; found C 47.92, H 3.88, N 10.81.

The elemental analysis shows that drying under vacuum results in the partial loss of the co-crystallized pyridine molecules in compounds **1** or **2**.

3. X-ray Crystallography.

Diffraction data were taken using an Oxford-Diffraction XCallibur S kappa geometry diffractometer (Mo-Kα radiation, graphite monochromator, λ = 0.71073 Å). To prevent evaporation of co-crystallised solvent molecules the crystals were coated with light hydrocarbon oil and the data were collected at 150 K. The cell parameters were obtained with intensities detected on three batches of 5 frames. The crystal-detector distance was 4.5 cm. The number of settings and frames has been established taking in consideration the Laue symmetry of the cell by CrysAlisPro Oxford-diffraction software.[2] 214 for **1** narrow data were collected for 1° increments in ω with a 110 s exposure time. Unique intensities detected on all frames using the Oxford-diffraction Red program were used to refine the values of the cell parameters. The substantial redundancy in data allows analytical absorption correction with Abspack Oxford-diffraction program.[2] Space groups were determined from systematic absences, and they were confirmed by the successful solution of the structure. The structures were solved by direct methods using the SHELXTL 6.14 package.[3] All non-hydrogen atoms were found by difference Fourier syntheses and refined on F^2. Hydrogen atoms were fixed in ideal position. Experimental details for X-ray data collections of **1** are given in Table S1. CCDC- 956785 contains the supplementary crystallographic data for this paper. These data can be obtained free of charge from The Crystallographic Data Centre via www.ccdc.cam.ac.uk/data_request/cif. Figure Graphics are generated using MERCURY 2.3 Supplied with Cambridge Structural Database; CCDC: Cambridge, U.K., 2004-2009.

---

[2] CrysAlisPro CCD, CrysAlisPro RED , ABSPACK, CrysAlis PRO. Agilent Technologies, Yarnton, England., **Agilent (2010)**.

[3] G. M. Sheldrick, 6.14 ed. (Ed.: B. A. Inc), Madison,Wisconsin, USA, **1997**.

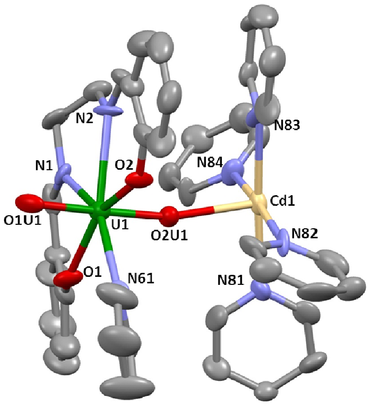

Figure *S1*. Mercury view of the coordination environment of U1 and Cd1 in **1** with thermal ellipsoids at the 50% probability level. Hydrogen atoms were omitted for clarity

Table *S1*. Crystallographic data of **1**

	**1**.2Py
Formula	$C_{51}H_{49}CdN_{10}O_7U$
Crystal size (mm)	0.23 x 0.14 x 0.04
cryst syst	Monoclinic
space group	P 2_1/c
volume (Å^3)	15084.9(15)
a (Å)	20.6165(13)
b (Å)	21.7877(12)
c (Å)	33.9309(17)
α (deg)	90
β (deg)	98.215(6)
γ (deg)	90
Z	12
formula weight (g/mol)	1264.43
density (g cm^{-3})	1.670
absorption coefficient (mm^{-1})	3.701
F(000)	7452
temp (K)	150.0(2)
total no. reflections	54830
unique reflections [R(int)]	25648 [R(int) = 0.1159]
Final R indices [I > 2σ(I)]	R1 = 0.0985, wR2 = 0.1845
Largest diff. peak and hole (e.Å^{-3})	2.730 and -2.160e.Å^{-3}
GOF	1.067

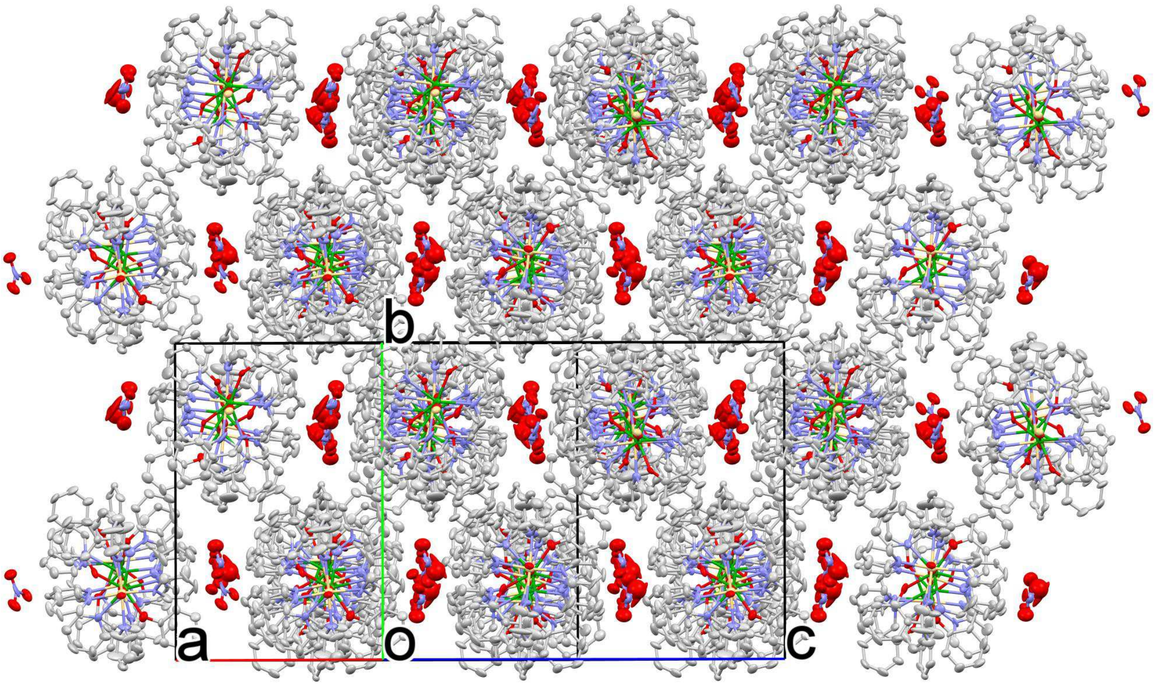

Figure *S2* Cell packing of complex **1**.2Py showing the alternating layers formed by the cationic polymeric chains and the nitrate anions (cocrystallized pyridine are removed for clarity).

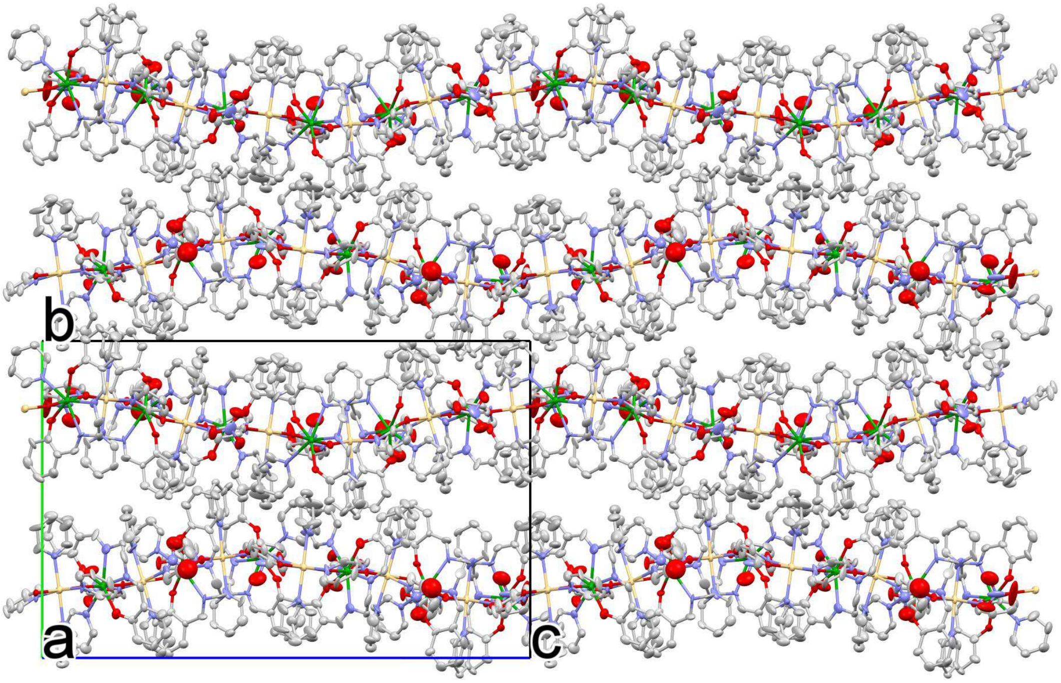

Figure *S3*. Mercury Diagram showing the non parallel arrangement of the cationic chain in **1** (cocrystallized pyridine are removed for clarity).

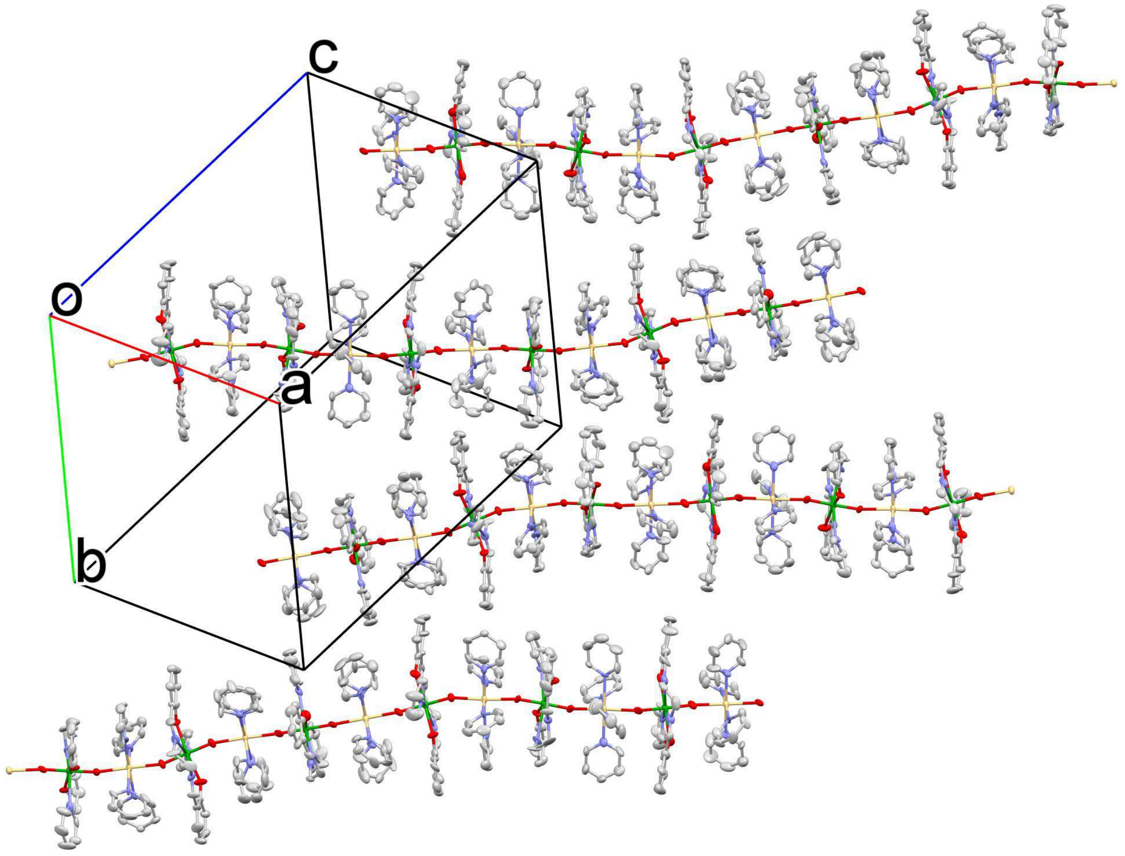

Figure *S4*. Cell packing of complex **1**.2py (cocrystallized pyridine and nitrate anions are removed for clarity).

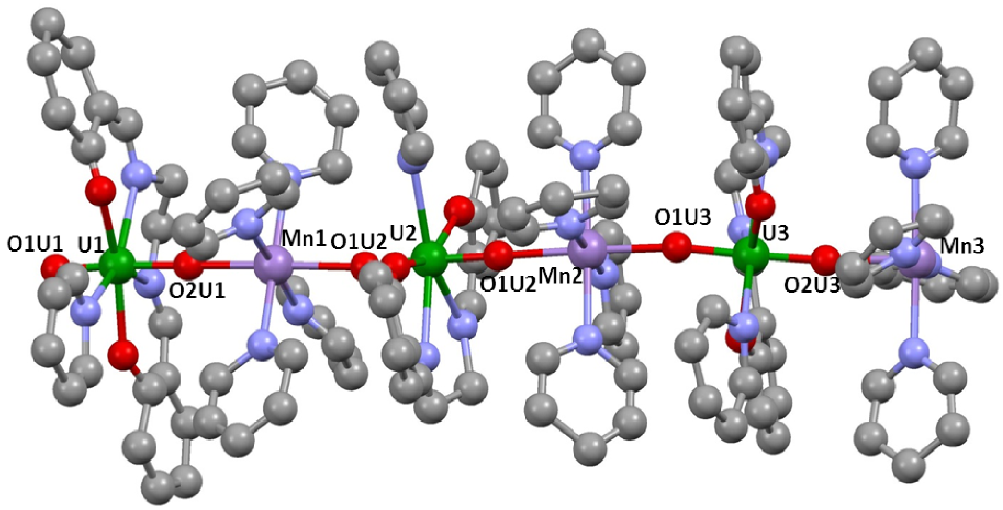

Figure *S5*. Mercury ball and sticks view of **2.** X-ray analysis shows that complex 2 is isostructural with complex **1** (cell parameters: 20.402(2); 21.776(3); 33.642(5); 90; 99.85(1); 90 ; space group $P2_1/c$). The high R value of 13.8% (due to the poor crystal quality) does not allow a detailed discussion of metrical parameters.

4. Small angle powder X-Ray diffractograms.

Powder diffraction measurements at small angle used a laboratory point collimation SAXS camera equipped with a rotating anode generator (FR591 Nonius) operating with a copper target at 45 kV and 66 mA and a point focus source of 200 μm size. The X-ray beam was Kα/Kβ filtered (λ=1.5418 Å) by total reflection from two curved Ni-coated Franks mirrors (Charles Supper Company) and by transmission through a thin 10 μm Ni foil. The low-angle X-ray diffraction patterns were recorded on a multi-wire gas filled proportional counter with a large 17x17 cm^2 active area which enables measurements covering a large range of scattering angle 2θbetween 0.6° and 30°. The sample-detector distance was 28 cm. The 2θ calibration of the low-angle X-ray diffraction instrument was performed using the scattering from a silver behenate sample with the first 11 (00l) diffraction peaks resulting from the (00l) long period spacing of 58.38 Å. The X-ray diffraction patterns of the powder samples were obtained by reduction of the two-dimensional data by radial integration of intensities from the center of the direct beam after data correction for the background scattering from the empty capillary. The samples were studied in 1mm quartz capillaries filled under argon in an inert glove box and then sealed.

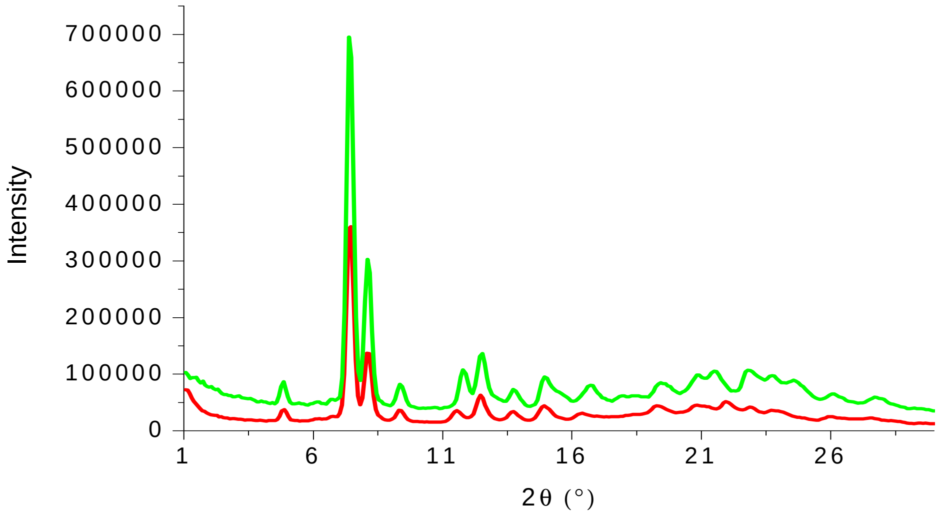

Figure *S6*. Small angle X-ray diffractogram of bulk compound **1** $\{[UO_2(salen)py][Cd(py)_4]NO_3\}_n$3.2(py) (red line) and **2** $\{[UO_2(salen)py][Mn(py)_4]NO_3\}_n$0.5(py) (green line).

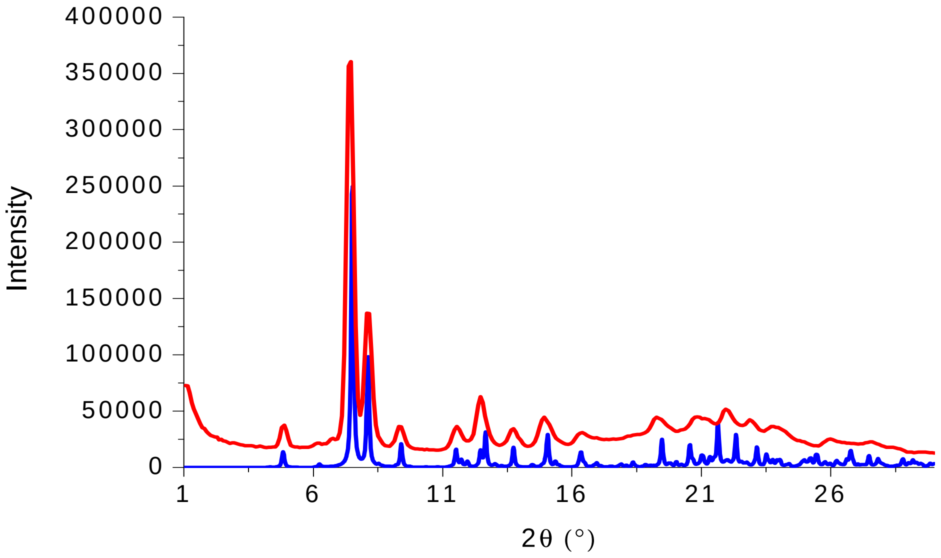

Figure *S7.* Small angle X-ray diffractogram of bulk compound **1** $\{[UO_2(salen)py][Cd(py)_4]NO_3\}_n$3.2(py) (red line) and diffractograms calculated from single crystal structures **1** $\{[UO_2(salen)py][Cd(py)_4]NO_3\}_n$.2(py) (blue line).

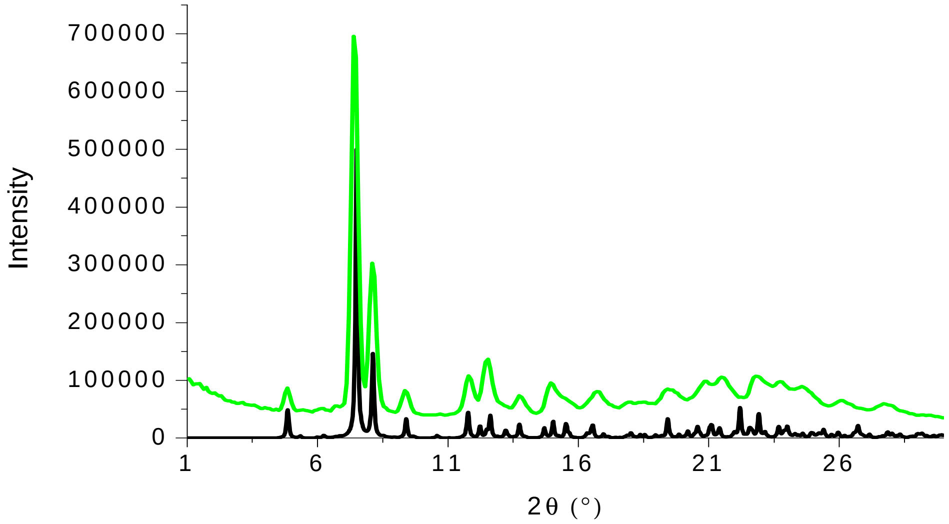

Figure *S8.* Small angle X-ray diffractogram of bulk compound **2** $\{[UO_2(salen)py][Mn(py)_4]NO_3\}_n$.0.5(py) (green line) and diffractograms calculated from single crystal structures of **2** (black line).

5. Magnetic studies.

Variable-temperature magnetic susceptibility and isothermal magnetization versus field data were recorded in the temperature range 2 – 300 K with a Quantum Design MPMS-XL 7T and a Quantum Design MPMS-XL 5.0 Superconducting Quantum Interference Devices (SQUID). Measurements were carried out on finely ground polycrystalline samples of **1** and **2** restrained in eicosane to prevent sample torquing. Plexiglas or Suprasil-Quartz tubes were used as sample holders and were sealed under argon before measurement.Reproducibility of the magnetic measurement was checked by the independent measurement of three samples from three different synthetic batches. The contribution to the signal of the empty sample holder was measured and subtracted from the total signal. Experimental data were further corrected for the diamagnetic contribution of the compounds using tabulated Pascal's constants. Magnetic calibration was done with a cylindrical palladium standard having approximately the same geometry of the measured samples.

Isothermal magnetization loops have been measured while sweeping the magnetic field at a constant rate, from 7 to -7 T and back, with the sample kept at different temperatures from 2 to 10 K.

The real and imaginary components, χ' and χ'', of the complex a.c. magnetic susceptibility were measured at zero-dc field as a function of temperature and frequency with a Quantum Design PPMS-14T platform using the mutual-inductance technique and a MPMS XL7 SQUID magnetometer.

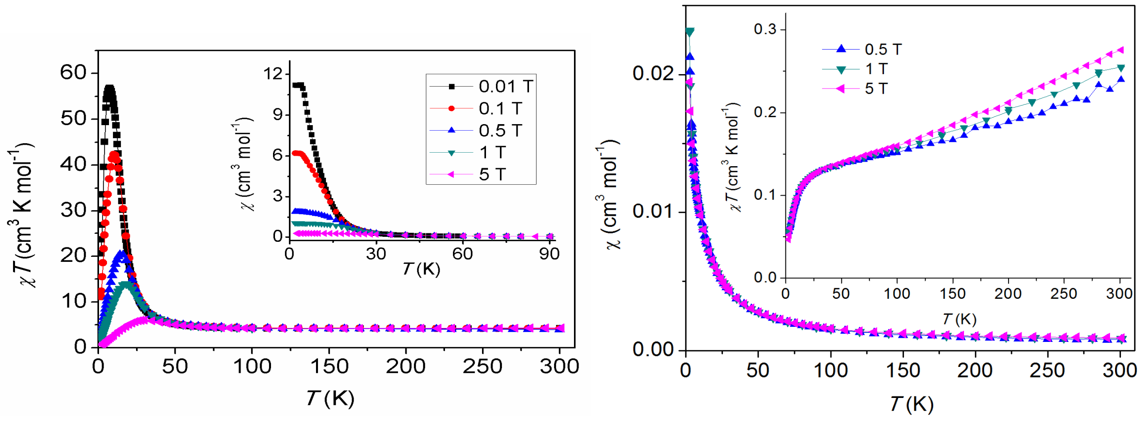

Figure *S9*. (left panel) Temperature dependence of χT for **2** measured at five different fields between 0.01 and 5 T. Inset: χ against T from 2 to 90 K measured at similar fields. (right panel) Temperature dependence of the magnetic susceptibility χ as a function of temperature T for **1** measured at three different fields between 0.5 and 5 T. Inset: Temperature dependence of χT for the same fields.

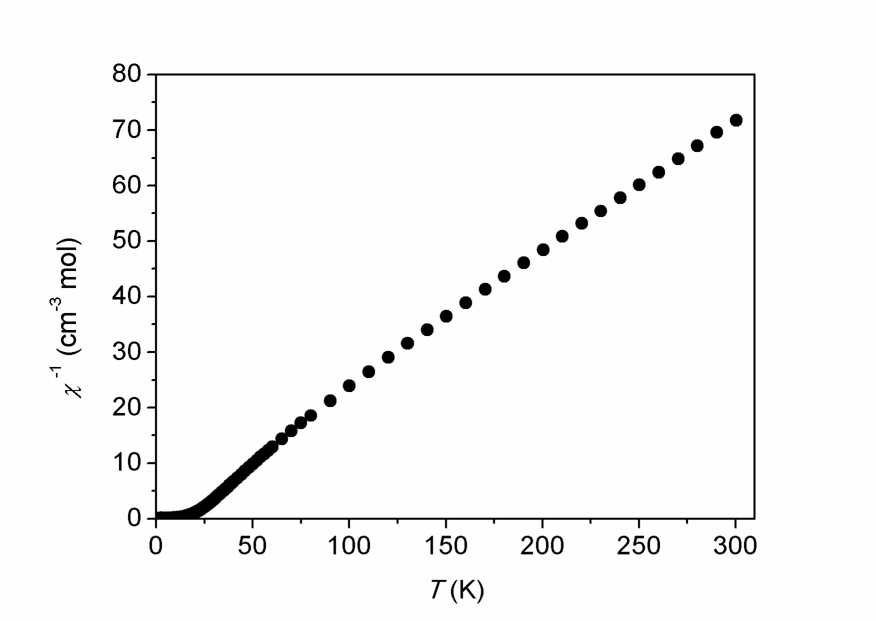

Figure *S10*. Temperature dependence of 1/χ for **2** measured at 0.01 T.

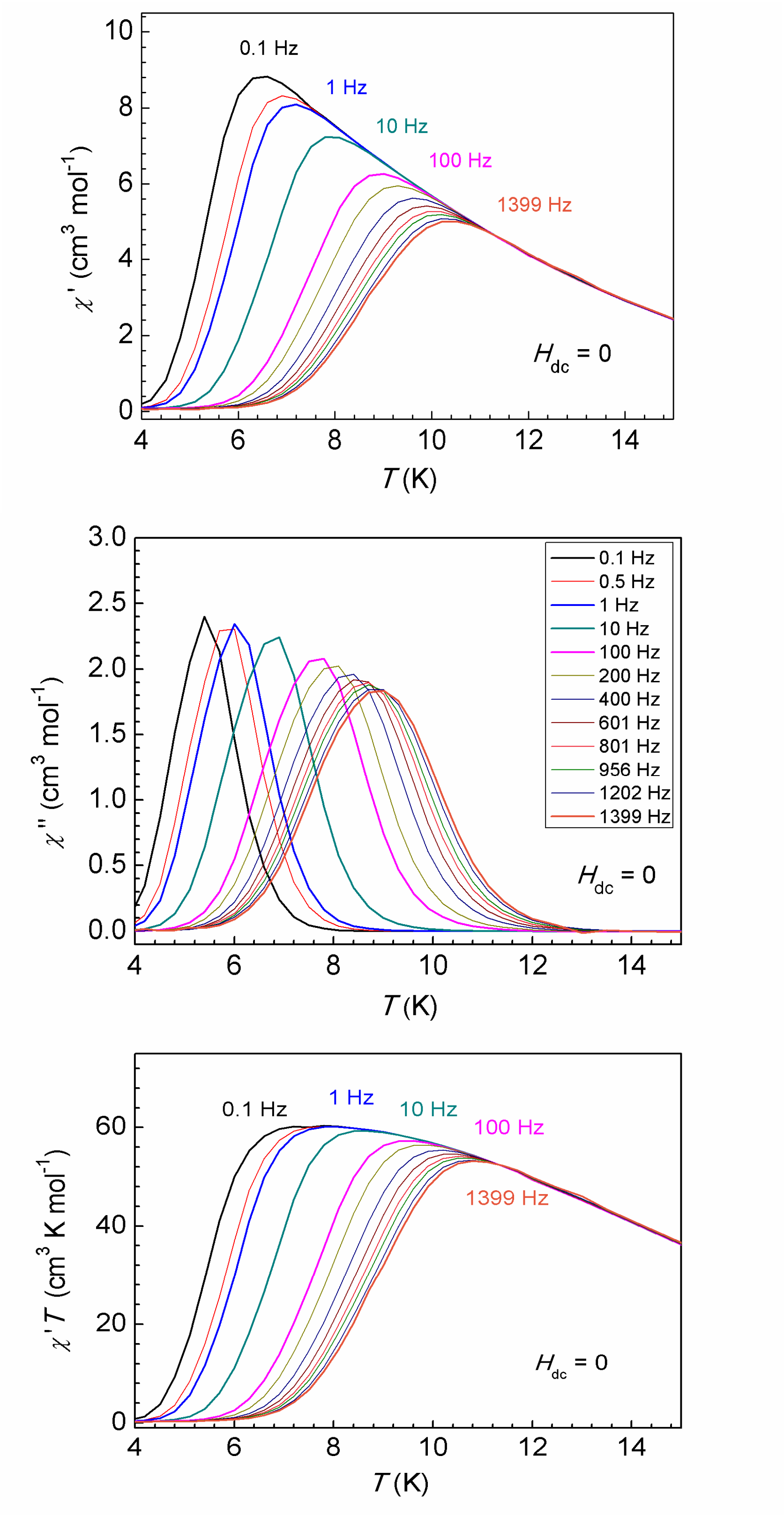

Figure *S11*. Temperature dependence of the ac magnetic susceptibility for **2** measured at zero dc field and an ac field of 1.55 G oscillating at frequencies between 0.1 and 1400 Hz.

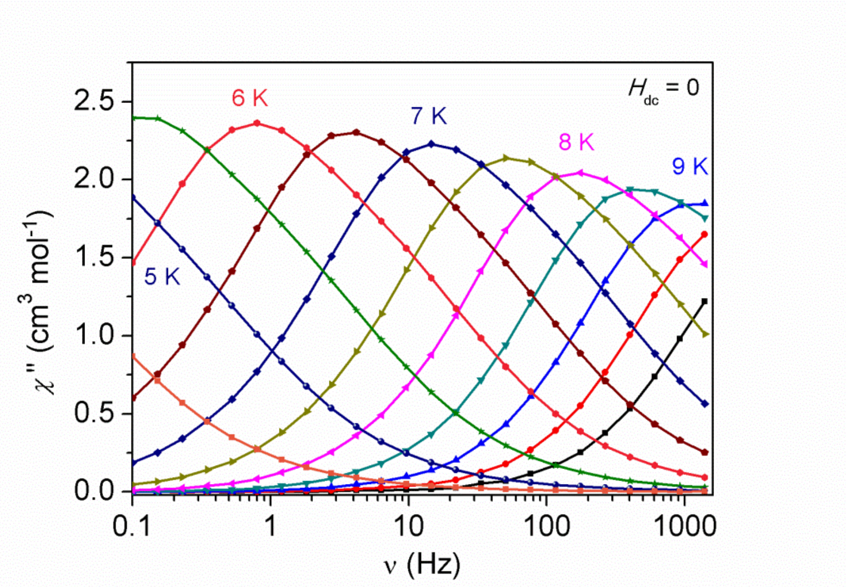

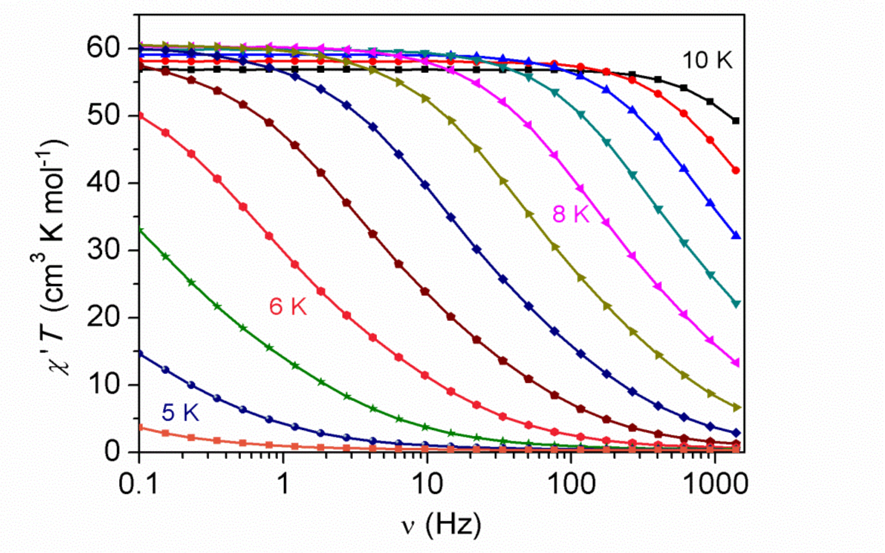

Figure *S12*. Frequency dependence of the ac magnetic susceptibility for **2** measured at zero dc field and an ac field of 1.55 G oscillating at frequencies between 0.1 and 1400 Hz.

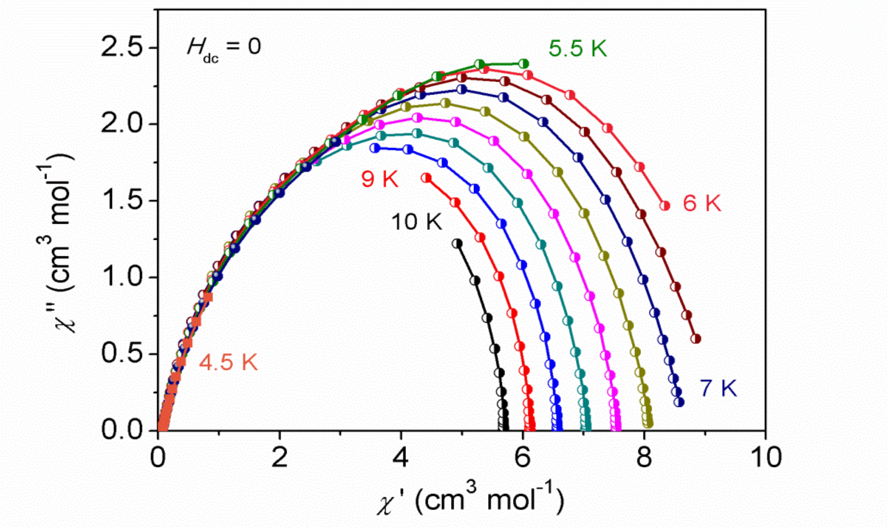

Figure *S13*. Cole-Cole plots for **2** measured at zero-dc field and an ac field of 1.55 G oscillating at frequencies between 0.1 and 1400 Hz.

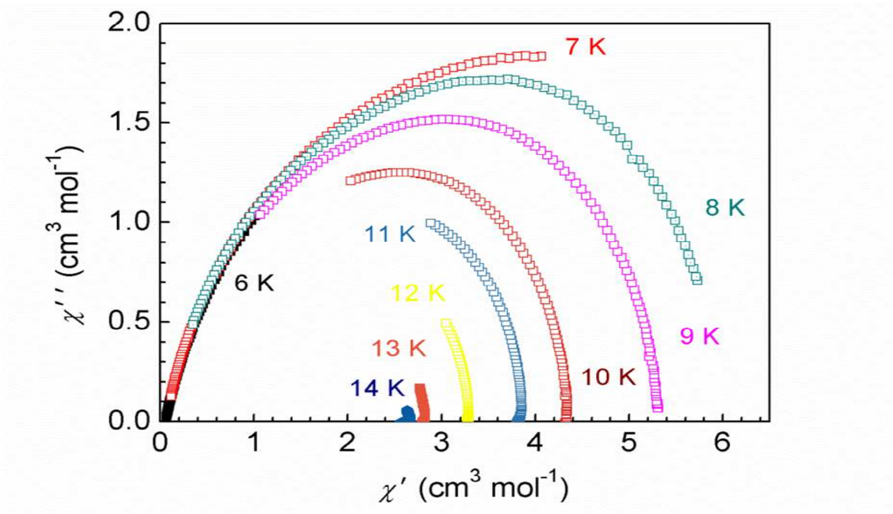

Figure *S14*. Cole-Cole plots for **2** measured at zero-dc field and an ac field of 10 G oscillating at frequencies between 10 and 9987 Hz.

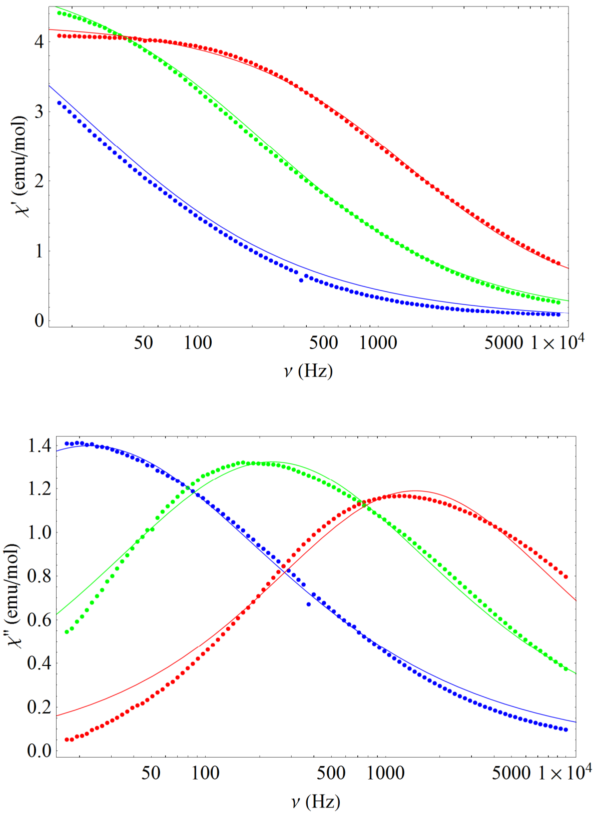

Figure *S15*. Data (dots) and fits (lines) for the ac susceptibility curves of **2** measured at 7 (blue), 8 (green) and 9 K (red). The modified Debye model described in detail in Ref. 9(c) has been used. The extracted values of the relaxation times τ for the three given temperatures are 7.0×10^{-3} s, 6.8×10^{-4} s, and 1.1×10^{-4} s respectively; the exponents α, which describe the relaxation time distribution, is 0.43, 0.39, and 0.33 respectively. The curves have been measured at zero-dc field and an ac field of 10 G oscillating at frequencies between 10 and 10000 Hz.

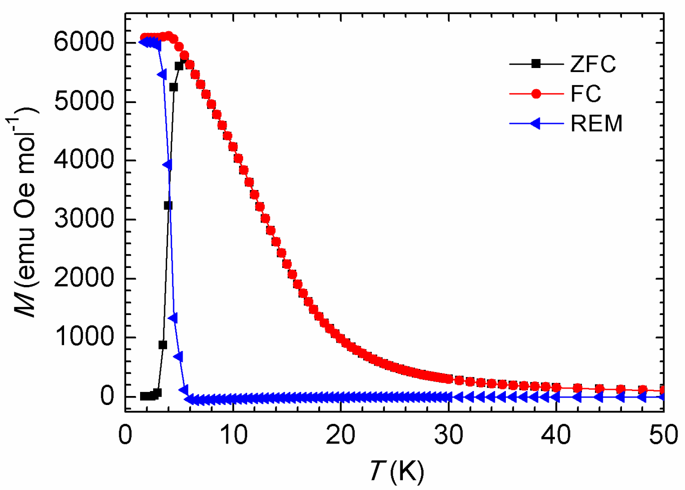

Figure *S16*. Temperature dependence of the zero-field cooled (ZFC), field-cooled (FC) and remanant (REM) magnetizations of **2** measured at 0.01 T.

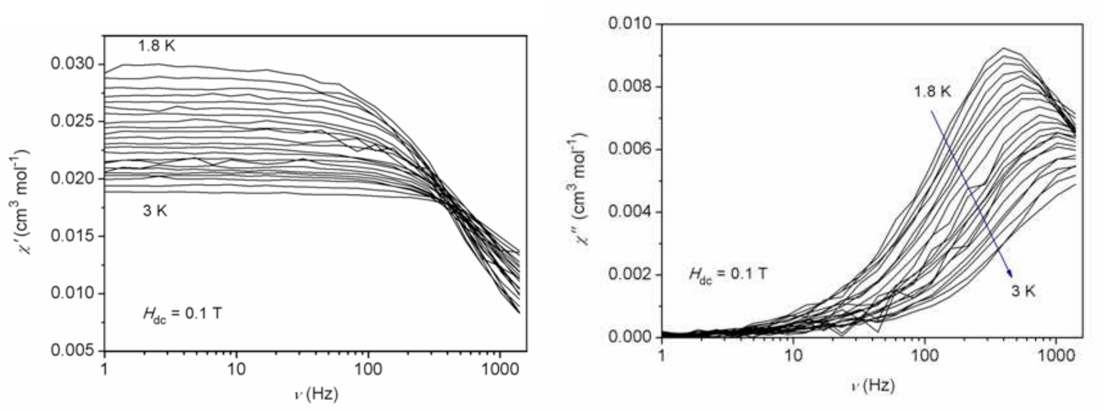

Figure *S17*. Frequency dependence of the (**left**) in-phase and (**right**) out-of-phase ac suspectibilities of **1** measured at 0.1 T dc field and 1.55 G ac field

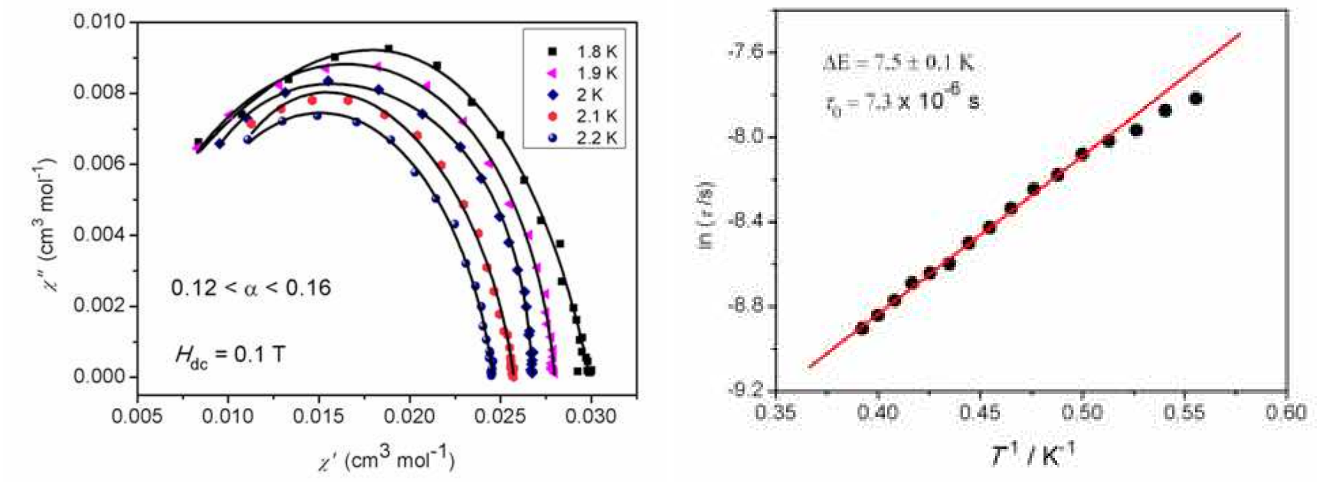

Figure *S18*. (Left) Cole Cole plots for **1** at temperatures between 1.8 amd 2.2 K; (right) Arrhenius plot displaying T-dependence of the relaxation time for **1**.
